# Perioperative pain management interventions in opioid user patients: an overview of reviews

**DOI:** 10.1186/s12871-024-02703-6

**Published:** 2024-09-05

**Authors:** Ava Tavakoli Vadeghani, Margaret Grant, Patrice Forget

**Affiliations:** 1 School of Medicine, Medical Sciences and Nutrition, University of Aberdeen, Aberdeen, UK; 2 Aberdeen Centre of Musculoskeletal Health (Epidemiology Group), Institute of Applied Health Sciences, School of Medicine, Medical Sciences and Nutrition, University of Aberdeen, Aberdeen, UK; 3 Department of Anaesthesia, NHS Grampian, Aberdeen, UK; 4 Pain and Opioids after Surgery (PANDOS) Research Groups, European Society of Anaesthesiology and Intensive Care, Brussels, Belgium; 5 IMAGINE UR UM 103, Anesthesia Critical Care, Emergency and Pain Medicine Division, Montpellier University, Nîmes University Hospital, Nîmes, 30900 France; 6 Institute of Applied Health Sciences, Epidemiology group, School of Medicine, Medical Sciences and Nutrition, Department of Anaesthesia, University of Aberdeen, NHS Grampian, Aberdeen, AB25 2ZD UK

**Keywords:** Pain management, Opioids, Acute pain, Surgery

## Abstract

**Background:**

Every year, many opioid users undergo surgery, experiencing increased postoperative complications, inadequate pain control, and opioid-related adverse effects. This overview aims to summarise and critically assess the systematic reviews about perioperative pain management interventions, identify the knowledge gaps, and potentially provide high-quality recommendations to improve postoperative analgesia and surgical outcomes.

**Methods:**

A systematic search was conducted from the following databases, PubMed, Cochrane Database of Systematic Reviews, Embase, APA PsycINFO, CINAHL, AMED, Scopus, PROSPERO, ProQuest, and Epistemonikos, in June 2023. Additionally, reference lists were reviewed. The identified studies were assessed based on eligibility criteria and data extracted by a self-designed form and two independent reviewers. Qualitative data were synthesised, and all included studies were assessed by The Assessment of Multiple Systematic Reviews 2 (AMSTAR 2) checklist.

**Results:**

Nine studies were included. The methodological quality of the studies was mostly critically low. Various interventions were identified, including perioperative management of buprenorphine, ketamine administration, multimodal analgesia, higher doses of medications, patient education, and interprofessional collaboration. The level of certainty of the evidence ranged from very low to high. One high-quality study showed that ketamine administration may improve perioperative analgesia supported with moderate to very low-quality evidence, and low and critically low studies indicated the efficacy of perioperative continuation of buprenorphine with low to very low-quality evidence.

**Conclusion:**

Perioperative continuation of buprenorphine and ketamine administration as a multimodal analgesia approach, with moderate to very low-quality evidence, improves pain management in opioid users and decreases opioid-related adverse effects. However, high-quality systematic reviews are required to fill the identified gaps in knowledge.

## Introduction

### Background

 Chronic opioid users consist of patients with opioid use disorder (OUD) on medication treatment (MOUD) such as buprenorphine, methadone, and naltrexone, those without pharmacological treatment, and patients who use prescribed opioids for chronic pain [1]. International Statistical Classification of Disease and Related Health Problems, 11th revision (ICD-11) suggests Disorders Due to Use of Opioids definition, including Opioid Dependence [2]. However, OUD is the preferred terminology by the Diagnostic and Statistical Manual-5 (DSM-5) [3]. Additionally, the O-NET classification system defines preoperative opioid tolerant as patients who used ≥ 60 mg morphine equivalent dose within seven days before the surgery [4].

In 2019, 8.3 million people were identified with illicit drug use and 1.6 million with prescription analgesic use disorder in the US [5]. Additionally, 310 million patients undergo surgery yearly [6] which 4-23% are chronic opioid users [7, 8]. Preoperative chronic use of opioids is associated with an increased risk of postoperative complications, such as respiratory failure, surgical site infection, induced mental disorder, readmission, and increased costs [7, 9–11]. These patients experience higher acute postoperative pain levels [12] and increased risk of postoperative chronic pain [13]. Even chronic administration of low-dose opioids may induce hyperalgesia and increase postoperative opioid consumption [14]. There are several guidelines to enhance surgical outcomes and pain management; however, There is a need to continually update existing guidance on this complex topic when high-quality evidence becomes available.

### Aim

This overview of systematic reviews summarises and critically assesses the quality of systematic reviews related to perioperative pain management interventions in opioid users. It also aims to identify knowledge gaps to help future research and possibly provide a list of high-quality recommendations for clinical practice to optimise pain management and surgical outcomes.

## Methods

### Review design

This overview of reviews was conducted based on the Reporting guideline for overviews of healthcare interventions: the Preferred Reporting Items for Overviews of Reviews (PRIOR) statement [15]. Ethics approval was not required for this literature-based project. Also, a predetermined protocol could not be registered in PROSPERO based on methodological criteria.

### Eligibility criteria

The eligibility criteria are shown in Table 1. In this overview, a systematic review was defined as any review that conducted a systematic search strategy and the authors mentioned it within their papers.

**Table 1:** Summary of eligibility criteria

Study Criteria	Inclusion Criteria	Exclusion Criteria
Study design	Systematic review and scoping reviews (+/- meta-analysis)	Non-reviews, protocols, narrative reviews, and other types of reviews which did not use a systematic search strategy
Population	Opioid users undergoing surgery (+/- opioid use disorder (OUD) treatment) and opioid use as a treatment of pain/ long-term opioid therapy (LTOT) (cancer and non-cancer pain)	No usage of opioids chronically; opioids usage for a brief time (acute use)
Intervention	Pharmacological and non-pharmacological interventions related to pain management	No limitations
Comparator	Any comparator including placebo, none, etc.	No limitations
Timing	Studies published in all years	No limitations
Outcome	An outcome related to perioperative pain management	An outcome unrelated to perioperative pain management (chronic and other types of pain)

This table summarises the inclusion and exclusion criteria of this overview. +/-: with or without item

### Search strategy

The search strategy involved the following databases: PubMed, Cochrane Database of Systematic Reviews, Embase, APA PsycINFO, CINAHL, Allied and Complementary Medicine (AMED), Scopus, PROSPERO/International Prospective Register of Systematic Reviews, ProQuest Dissertations & Theses A&I, and Epistemonikos, from inception until June 2023. The results were limited to English language and systematic review study design. Google Scholar was also hand-searched for related systematic reviews. The search included “opioid users” and “perioperative pain management” keywords. The details of the search strategy for each database are provided in Appendix 1. Furthermore, the reference lists of included studies were reviewed.

### Study selection

Rayyan AI [16] was used to manage the studies. Duplicates were automatically detected, manually screened, and deleted. Two independent reviewers screened the results by titles and abstracts. Irrelevant studies were eliminated. Then, the full text of the studies was screened based on eligibility criteria. Any questions or uncertainties were addressed and resolved through discussion and consensus among the two reviewers and third one.

### Data collection

A self-designed data extraction form was used to manually collect data from included studies. This form consisted of the following items: author, title, year of publication, country, search period, number of primary studies included, total number of participants, aim, population, intervention, primary outcomes, study designs, funding sources, quality evaluation method, degree of certainty, conduct of meta-analysis (yes/no), study limitations, areas for future research, and main findings. Data collection was done by two reviewers independently and any disagreement was resolved by consensus. Data collection was done by two reviewers independently and any disagreement was resolved by consensus.

### Quality assessment

The Assessment of Multiple Systematic Reviews 2 (AMSTAR 2) checklist was used to assess the methodological quality of the reviews included by two independent reviewers [17]. This checklist consists of 16 items (Appendix 2) and presents the overall rating based on weaknesses in the critical domain in the form of the following categories, high (≤ 1 non-critical weakness), moderate (> 1 non-critical weakness), low (1 critical flaw with or without non-critical weaknesses), critically low (> 1 critical flaw with or without non-critical weaknesses). The authors of the included reviews were contacted to provide complementary data. The results were presented with all questions and overall ratings in a table.

### Data synthesis

The data were synthesised qualitatively and presented as a summary table. The interventions used by each review were extracted and categorised, and a narrative synthesis of the results was conducted.

## Results

### Study selection

**Fig. 1:**
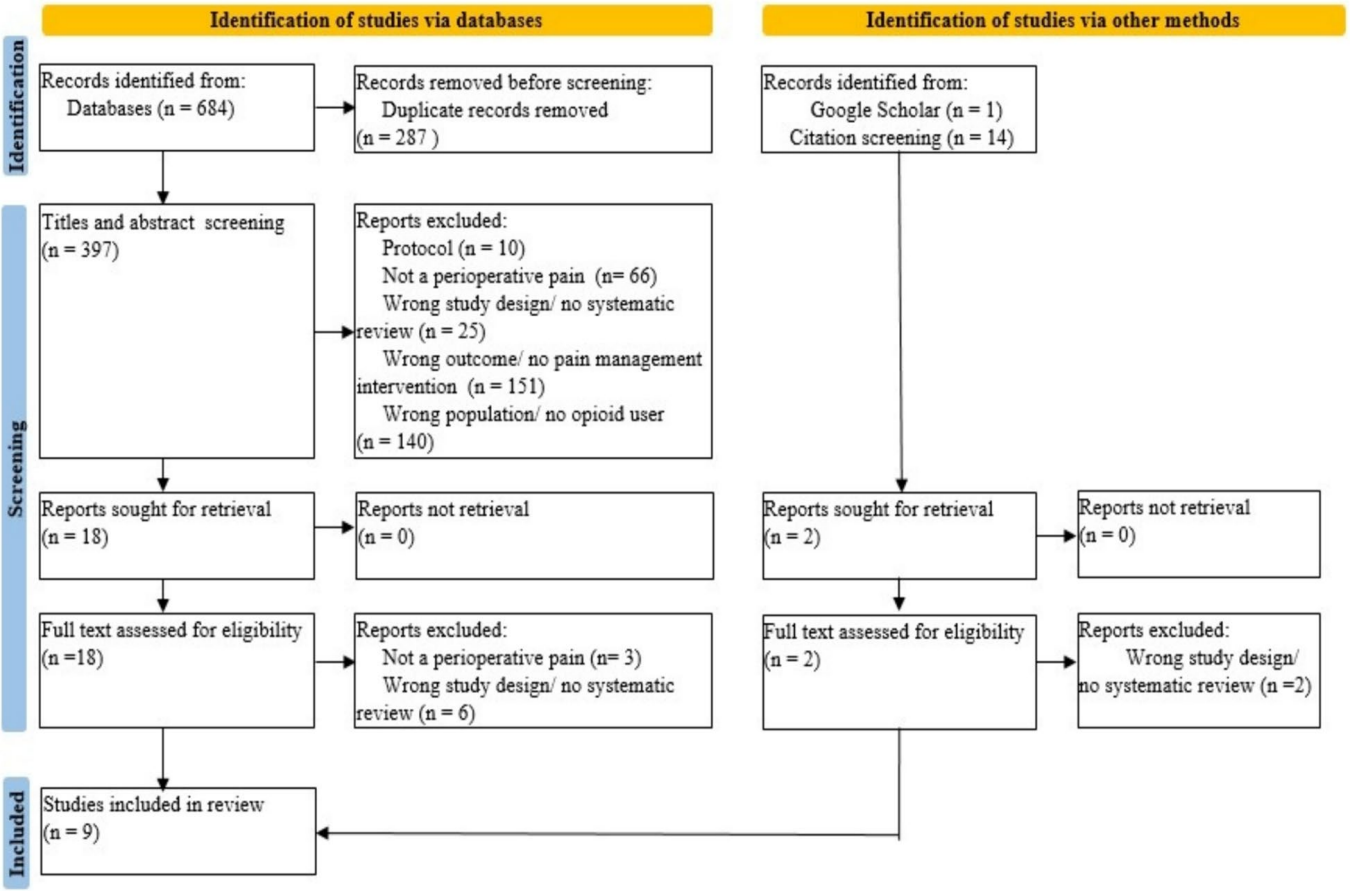
PRISMA flow diagram. This figure demonstrates the study selection process based on the Preferred Reporting Items for Systematic Reviews and Meta-Analyses 2020 flow diagram [18]

Electronic searches of ten databases were conducted, and ProQuest and APA PsycINFO had no results regarding the search strategy. In total, 699 studies were identified. After removing the duplicates and adding other identification methods results, 412 studies were screened with titles and abstracts. Three hundred and ninety-two studies were excluded (Fig. 1). Twenty studies were retrieved and screened in full text by eligibility criteria, and 11 studies were excluded. Three studies did not cover perioperative pain, and eight had the wrong study design (Appendix 3). Finally, nine studies were included in the overview (Table 2).

### Study characteristics

Most systematic reviews were conducted in the USA (*n* = 5), and only one European country (Germany) was identified [19]. While one study was conducted in 2014 [20], the remaining studies were published between 2019 and 2022. Additionally, two studies did not report the search date or their last update [4, 21]. Only Meyer-Frießem et al. conducted a meta-analysis [19], and three of the studies were scoping reviews that used systematic search strategies [20, 22, 23]. Furthermore, Edwards et al. and Quaye et al. used their reviews to identify available studies associated with perioperative pain management interventions followed by consensus recommendations [4, 23]. Against inclusion criteria, Veazie et al. included all causes of acute pain; however, 66.7% of their included studies were exclusively focused on perioperative pain management, and the remaining covered non-specified acute pain [24]. Mehta et al., Edwards et al., and Veazie et al. restricted their populations to adults (≥ 18 years) [4, 24, 25], and Lim et al. only investigated pregnant patients [22]. In terms of opioid user definition, Meyer-Frießem et al. and Edwards et al. included all opioid users [4, 19]. Four studies restricted their reviews to patients on MOUDs, particularly buprenorphine [23–26]. One study only included randomised clinical trials (RCTs), while others included any designs. Four reviews included various interventions. Others were more specific, with one study focused on perioperative ketamine administration and four studies comparing the continuation and discontinuation of buprenorphine, one of which also included other MOUDs modifications. The reviews included 9–84 studies, and only two reported the total number of participants [19, 25]. The studies checked for various and heterogeneous outcomes. Some of them did not mention their outcomes clearly. However, most reviews considered the adverse effects and efficacy of interventions via scoring pain, opioid consumption, and risk of OUD.

**Table 2:** Studies characteristics

Author Year Country	Search Period	Population	Interventions	Eligible Study Designs	*N* Studies/Total Patients/RCTs	Primary Outcomes
Buckley [20] 2014 Canada	Up to April 2013	Patients with OUD, treated with MOUD, untreated or on abstinence-based treatment undergoing surgery or obstetrical care	Perioperative pain management	Any designs	27 / NR	Any obstetric care and perioperative management intervention
Goel [26] 2019 Canada	Up to June 2017	Patients on buprenorphine for either chronic pain or OUD undergoing surgery	Perioperative buprenorphine management: continuation or discontinuation	Any designs	18 / NR	Relative effectiveness of the interventions and reporting complications, pain parameters, and long-term follow-up if available (opioid relapse and chronic pain)
Quaye [23] 2019 USA	Up to March 2018	Patients on buprenorphine for OUD undergoing surgery	Perioperative buprenorphine management: continuation or discontinuation	Any designs	12 / NR	Postoperative pain management strategies and complications including the risk of relapse and opioid consumption
Edwards [4] 2019 USA	NR	Adult chronic opioid users undergoing surgery	Perioperative pain management	Any designs	50 (32 for pain management)/ NR	The impact of any interventions aimed toward the care of patients
Mehta [25] 2020 Canada	Up to January 2019	Adults on buprenorphine for OUD undergoing surgery under general anesthesia	Perioperative buprenorphine management: continuation or discontinuation with or without bridging to another mu-opioid agonist	Any designs	18 / 202	Postoperative pain intensity, total opioid use, and identification of benefits and harms of perioperative strategies
French [21] 2020 USA	NR	Chronic opioid users undergoing surgery	Perioperative nursing/ care	Any designs	25 / NR	Any perioperative care/ nursing included and needed and knowledge gap
Veazie [24] 2020 USA	Up to April 2020	Nonpregnant adults on MOUD who have acute (sudden onset, time-limited) pain	Perioperative pain management	Any designs	12 / NR	Pain severity, pain-related function, quality of life, patient satisfaction, healthcare utilization, opioid withdrawal symptoms, substance use relapse, opioid overdose, suicidal ideation, suicidal self-directed violence, and other adverse events
Lim [22] 2022 USA	Up to March 2020	Pregnant people with OUD, both treated with MOUD and untreated	Peridelivery pain management	Any designs	84 / NR	Knowledge gap and peridelivery pain management interventions
Meyer-Frießem [19] 2022 Germany	Up to July 2020	Adult opioid users undergoing surgery	Perioperative administration of ketamine	RCTs	9 / 802	Postoperative pain at rest and during movement 24 h after surgery and the number of patients with any ketamine-related adverse event

This table summarises the characteristics of included reviews and uses the first author's name. In the RCTs section, in cases where systematic review included RCTs, "Yes" has been used. *NR* Not Reported, *OUD* Opioid Use Disorder, *MOUD* Medication for Opioid Use Disorder, *RCTs* Randomized Controlled Trials, *N* Number

### Risk of bias in the reviews

Less than half of the reviews (4/9) reported their risk of bias or quality of evidence assessment. Meyer-Frießem et al. used Cochrane’s Risk of Bias 2 (ROB2.0) and reported the results in detail. Most of their primary studies had a high risk of bias and only one had uncertain risks. They also used the Grading of Recommendations Assessment Development and Evaluation (GRADE) for quality of evidence assessment and reported the following results: moderate (*n* = 1), low (*n* = 3), and very low (*n* = 4) quality [19]. Edwards al. also employed GRADE and reported the results with A to C, levels one to four, and moderate to very low [4]. French et al. used the Study Quality Assessment Tools of National Heart, Lung, and Blood Institute (NHLBI) that resulted in good to poor quality primary studies (good: *n* = 8, fair: *n* = 2, poor: *n* = 1). The authors only reported the assessment of 44% of studies (11/25) [21]. Veazie S et al. employed Cochrane’s Risk Of Bias In Non-randomized Studies of Interventions (ROBINS-I) tool, modified with the CAse REport (CARE) checklist for observational studies without control groups. Three of their primary studies had a high risk of bias, nine were partly reported, and one was mostly reported regarding the quality of reporting of evidence [24].

### Quality of evidence

The AMSTAR2 checklist was used for the quality of evidence assessment of included reviews [17]. Most reviews had critically low quality (7/9 studies) [4, 20–23, 25, 26]. One study was rated as low quality [24], and only one study achieved high methodological quality [19]. Despite emailing eight authors to provide more data, none of them responded. None of the studies contained all AMSTAR2 items. Almost all authors defined PICO adequately and explained their rationale behind study selection [4, 19, 21–26]. However, one study did not mention the comparators in the eligibility criteria [20]. While all the authors declared their funding sources and conflicts of interest, none of them reported the funding sources of their primary studies. Most authors comprehensively reported the literature search strategy; however, searching for grey literature and clinical trials, using experienced consultants in the field, and conducting the search within 24 months of completing the review were not reported in many reviews. Only one of the authors reported the search strategy completely [24]. Moreover, most reviews selected and reviewed the studies in duplicate [4, 19, 20, 22, 24–26]. Only one author reported the excluded articles with the reason for exclusion [19]. Also, Items 2, 7, 9, 13, and 14 were not reported in most reviews (Table 3).

**Table 3:**
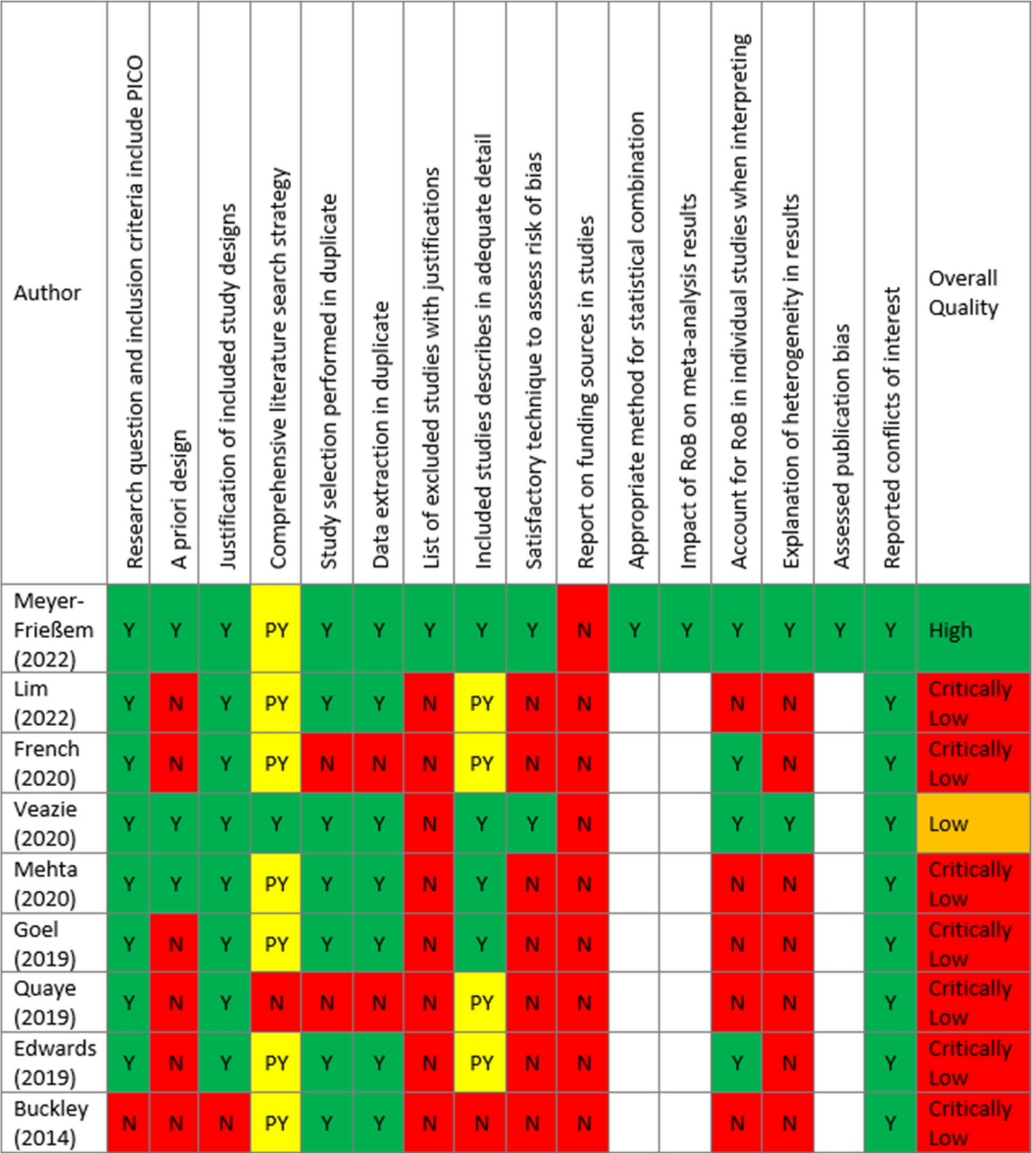
Quality assessment results [4, 19–26]

This table demonstrates the methodological quality of included reviews based on the Assessment of Multiple Systematic Reviews 2 (AMSTAR 2) tool [17]. In this table, the summarised form of the questions is shown. The full text of the questions is provided in Appendix 2. The empty cells identify that no meta-analysis is conducted by the authors. N: No; Y: Yes; PY: Partial Yes.

###  Main findings

#### Perioperative buprenorphine management

Four included studies focused on buprenorphine management, and three more investigated it as part of their review. All studies recommended continuing buprenorphine perioperatively, but two reviews suggested a reduced daily dose (Table 4). Goel et al. found no evidence supporting the harm reduction of buprenorphine discontinuation in the perioperative period. They concluded that if the daily dose of the sublingual form of medication is ≤ 16 mg, it can be continued without more harm. However, for patients with a higher risk of addiction relapse, discontinuation of buprenorphine should be assessed carefully based on patient and surgery considerations. Three studies reported reduced postoperative opioid consumption with buprenorphine continuation [22, 24, 26]. Quaye et al. showed that perioperative continuation of buprenorphine does not increase the risk of adverse effects, and patients who interrupted buprenorphine have a higher risk for postoperative OUD relapse, illicit opioid use, opioid withdrawal symptoms, and amplification of chronic pain. They recommended buprenorphine continuation with a reduced dose to optimise the analgesic effects of opioid agonists and prevent withdrawal symptoms and proposed an algorithm for major surgery [23]. Mehta et al. reported the range of buprenorphine daily dose 2–32 mg associated with various multimodal analgesia strategies. Their review identified that pain management in patients on MOUD is more challenging than without it [25]. Veazie et al. confirmed others’ findings and suggested that insufficient pain management may increase the risk of discontinuation of MOUD treatment [24]. Lim et al. emphasised the low quality of the studies and identified knowledge gaps (Table 5) [22]. There are overlaps of primary studies among Lim et al., Goel et al., and Mehta et al. reviews that resulted in similar findings and recommendations [22, 25, 26]. Buckley et al. and Edwards et al. also recommended continuation of MOUDs [4, 20].

**Table 4:** Summary of findings associated with continuation of buprenorphine perioperatively

Author	Findings	Recommendations	Quality Assessment
Goel (2019) [26]	• Adequate analgesia with continued buprenorphine ≤ 16 mg/d • Less postoperative opioid and more NSAIDs consumption	• Continuation of buprenorphine when daily SL dose is ≤ 16 mg/d • In patients with an elevated risk of relapse, buprenorphine discontinuation should only be considered if there are convincing reasons regarding patient preference and surgical considerations.	• Critically low
Mehta (2020) [25]	• More difficult postoperative pain control compared with those who are not on MOUD • No evidence supporting the preference for one postoperative analgesia strategy • Lack of clear advantage and elevated risk of failure to return to buprenorphine baseline doses, continuing opioid agonist use, or OUD relapse with buprenorphine discontinuation	• Continuation of buprenorphine perioperatively combined with multimodal analgesia when possible	• Critically low
Quaye (2019) [23]	• Adequate analgesia with receiving opioid agonists • No increased risk of adverse events • Increased risk of illicit opioid use, OUD relapse, withdrawal symptoms, and amplification of chronic pain with discontinuation of buprenorphine	• Continuation of buprenorphine perioperatively with reduced dose: Decreasing buprenorphine daily dose to 16 mg one day before surgery, then lowering it to 8 mg/d with an opioid agonist for the subsequent days.	• Critically low
Veazie (2020) [24]	• More difficult postoperative pain control compared with those who are not on MOUD • Less postoperative opioid consumption • Increased risk of discontinuation of the methadone for OUD treatment in inadequate pain management • Adequate analgesia with tramadol for patients on naltrexone	• Continuation of MOUDs for most patients	• Low
Lim (2022) [22]	• Adequate analgesia with receiving opioid agonists and NSAIDs • Less postoperative opioid and more NSAIDs consumption • Less requirement for postoperative opioids for patients on buprenorphine compared with methadone	• Continuation of MOUDs combined with multimodal analgesia and additional opioids in the peridelively period	• Critically low
Buckley (2014) [20]		• Continuation of MOUDs combined with multimodal analgesia and additional opioids in the peridelively period • Designing a strategy for tapering postoperative opioid with supportive approaches, including multimodal and regional analgesia in patients with abstinence-based treatment	• Critically low
Edwards (2019) [4]		• Continuation MOUDs perioperatively and increase in MOUDs doses or opioid agonist addition in the postoperative period	• Critically low

This table summarises the findings of the included reviews related to perioperative management of buprenorphine and has provided the authors’ recommendations regarding it. *SL* Sublingual, *NSAIDs* Non-Steroidal Anti-Inflammatory Drugs, *MOUD* Medication for Opioid Use Disorder

**Table 5:** Summary of areas for future research

Author	Areas for Future Research
Goel; Veazie; Meyer-Frießem	• Long-term outcomes, including morbidity and mortality
	• Outcomes: Rate of OUD relapse, patient satisfaction, and withdrawal symptoms
Buckley (2014) [20]	• Controlled trials on postpartum pain management interventions
Goel (2019) [26]	• Details of buprenorphine dose and route of administration
Edwards (2019) [4]	• Development of the ERAS protocols specific to the population
Quaye (2019)[23]	• The optimal dose of buprenorphine use in the perioperative period
French (2020) [21]	• Nurses’ role in pain management
	• Patients’ race and ethnicity role in the delivery of nursing care
	• Ways which improve patients’ education
Veazie (2020) [24]	• Nonopioid treatments for patients on naltrexone as MOUD
	• Prospective and high-quality studies of adjuvant analgesia strategies in continuation of MOUD
	• Efficacy of slow-release oral morphine in acute pain management for patients with OUD
Lim (2022) [22]	• Nonopioid and nonpharmacologic analgesia methods in the peridelivery period
	• Using opioids as rescue analgesics, their optimal dose, and monitoring techniques in the peridelivery period
	• Monitoring the adverse effects of coadministration of opioids with other analgesics, including respiratory depression and sedation in the peridelivery period
	• The optimal dose of neuraxial analgesia in the peridelivery period
	• Role of continuous wound infiltration and truncal nerve blocks for postpartum pain management
	• Optimal methods for psychosocial aspects of postpartum pain management
Meyer-Frießem (2022) [19]	• Prevalence of perioperative ketamine adverse effects, CNS-related
	• The optimal dose of perioperative ketamine and its treatment duration
	• Effects of perioperative ketamine on prevention and treatment of depression symptoms associated with chronic opioid use

This table is the summary of areas for future research identified and extracted from included reviews. OUD: Opioid Use Disorder; MOUD: Medication for Opioid Use Disorder; ERAS: Enhanced Recovery After Surgery

#### Perioperative administration of ketamine

Meyer- Frießem et al. investigated the efficacy of perioperative administration of ketamine (Table 6). They conducted a high-quality systematic review and meta-analysis; however, the quality of evidence regarding their outcomes was moderate to very low. They showed that perioperative administration of ketamine may decrease postoperative pain during the movement, opioid-related side effects, and total opioid consumption within 48 h after surgery. They recommended considering ketamine in the pain management strategies of opioid users. The range of ketamine doses was 0.15–0.5 mg/kg for bolus injection and 0.002 mg/kg/h-0.25 mg/kg/h for maintenance infusion [19]. Furthermore, French et al. recommended using ketamine infusion as a part of the multimodal analgesia approach in patients with methadone maintenance therapy [21]. Edwards et al. highlighted that the efficacy of ketamine is dependent on the dose of ketamine and the type of surgery [4].

#### General interventions

Multimodal analgesia has been recommended by most of the reviews as a combination of different approaches, including the administration of NSAIDs, paracetamol, dexamethasone, lidocaine, alpha2 agonists, gabapentinoids, and N-methyl-D-aspartate (NMDA) receptor antagonists [20–25]. Quaye et al. showed that these approaches improve analgesic efficacy [23]. Edwards et al. also concluded with a high degree of certainty that the multimodal analgesia approaches, a combination of opioid and nonopioid analgesics, regional analgesia, and nonpharmacological treatments, optimise pain management and reduce the associated adverse effects. However, they didn’t identify evidence supporting nonpharmacological treatments’ efficacy, including distraction therapy, music therapy, hypnosis, and transcutaneous electrical nerve stimulation. Additionally, Edwards et al. recommended that prescribing opioids should be conditional to insufficient pain management by nonopioid analgesics, and individualised minimum effective doses and tapering opioids collaborating with the patient’s outpatient provider should be considered [4]. This study also recommended weaning opioids preoperatively to the minimum effective dose based on the patient’s condition and its feasibility.

Moreover, the findings of French et al., Veazie et al., Lim et al., and Mehta et al. showed that patients who use opioids chronically required higher doses of analgesics to control postoperative pain effectively but only with low-quality evidence. [21, 22, 24, 25]. French et al. and Quaye et al. recommended an interprofessional collaboration among addiction and pain specialists, nurses, anaesthesiologists, surgeons, psychiatrists, and patients [21, 23]. Patient education and awareness of potential adverse effects and realistic postoperative pain also play critical roles in the effectiveness of pain control and managing patient expectations [4, 21, 23].

#### Pregnancy

Lim et al. and Buckley et al. focused on peridelivery pain management in opioid users [20, 22]. However, the primary studies had low-quality evidence, leading to numerous knowledge gaps (Table 5). Since most reviews used pregnant and caesarean cases for buprenorphine management, the detailed results and recommendations were mentioned in the corresponding previous sections.

**Table 6:** Summary of findings associated with general interventions

Intervention	Author	Findings/Recommendations	Quality of Evidence	AMSTAR 2
**Multimodal analgesia** **(MMA)**	Buckley (2014) [20]	• Implementation of MMA strategies, including regional analgesia, ketamine infusions, NSAIDs, and paracetamol	NR	Critically low
	Quaye (2019)[23]	• Utilising adjuvant and opioid-sparing analgesia, including NSAIDs, gabapentinoids, alpha2 agonists, and NMDA receptor antagonists, to improve analgesic efficacy and limit opioid consumption	NR	Critically low
	Edwards (2019) [4]	• Implementation of individualised MMA strategies, including regional/neuraxial analgesia, nonopioid analgesics, and nonpharmacological treatments	High	Critically low
	Veazie (2020) [24]	• Utilising adjuvant analgesics, including NSAIDs, paracetamol, continuous ketamine infusion, and clonidine	NR	Low
	French (2020) [21]	• Considering the combination of regional nerve block, ketamine infusions, NSAIDs, paracetamol, dexamethasone, lidocaine, and mindfulness relaxation to enhance postoperative pain management	NR	Critically low
	Mehta (2020) [25]	• Implementation of MMA strategies, including epidural analgesia with fentanyl + postoperative morphine PCA or hydromorphone PCA + NSAIDs, excessive buprenorphine, SQ morphine, and fentanyl PCA	NR	Critically low
**Ketamine**	Edwards (2019) [4]	• Benefits are dependent on the dose of ketamine and the type of surgery	NR	Critically low
	French (2020) [21]	• Improvement of pain management in patients on methadone maintenance therapy	Low	Critically low
	Meyer-Frießem (2022) [19]	• Reduced postoperative pain during movement and total opioid consumption at 24 h • Any impact on postoperative pain during rest after 24 h, and adverse events, including hallucinations and confusion within 48 h • Reduced total opioid consumption within 48 h and relative risk of sedation induced by opioids	Low Very low Moderate	High
**Higher doses of medication**	Buckley (2014) [20]	• Requirement of higher opioid doses for postoperative analgesia	NR	Critically low
	Veazie (2020) [24]	• Requirement of higher doses for postoperative analgesia	Low	Low
	French (2020) [21]	• Requirement of higher doses for postoperative analgesia	NR	Critically low
	Lim (2022) [22]	• Requirement of higher opioid doses for postpartum analgesia	NR	Critically low
**Patient education**	Quaye (2019) [23]	• Education about patient expectations following surgery, including the typical time course for acute pain and realistic goals for pain management	NR	Critically low
	Edwards (2019) [4]	• Individualised preoperative education to improve pain management-related expectations	NR	Critically low
	French (2020) [21]	• Education about postoperative adverse effects to improve care delivery and patient experience	NR	Critically low
**Interprofessional collaboration**	Quaye (2019) [23]	• Collaboration among pain management specialists, addiction medicine specialists, and psychiatrists when it is necessary	NR	Critically low
	French (2020) [21]	• Collaboration between the patient, surgeons, nurses, anesthesiologists, addiction and pain management specialists, and primary care providers to optimise outcomes	NR	Critically low

This table summarises the general findings and recommendations identified from the reviews, including multimodal analgesia, ketamine administration, the need for higher doses of opioids and non-opioid analgesics, patient education, and interprofessional collaboration. The quality of evidence mentioned in the table is based on the authors' reports. *NR*  has been provided when the quality assessment was not conducted, or authors did not accurately report it. *MMA* Multimodal Analgesia, *PCA* Patient Controlled Analgesia, *NSAIDs* Non-Steroidal Anti-Inflammatory Drugs, *SQ* Subcutaneous, *NMDA* N-methyl-D-aspartate, *NR* Not Reported

## Discussion

### Main findings

This overview summarised the latest findings of systematic reviews associated with perioperative pain management interventions in the opioid user population. The review included several studies, with the majority having critically low methodological quality and only with high quality [19]. The level of certainty of the evidence ranged from very low to high. The high-quality review demonstrated that opioid users may benefit from perioperative administration of ketamine with moderate to very low-quality evidence [19]. Additionally, low and critically low systematic reviews revealed that perioperative continuation of buprenorphine may improve postoperative analgesic outcomes with very low to low-quality evidence [4, 20, 22–26]. Furthermore, critically low systematic reviews demonstrated the effectiveness of multimodal analgesia approaches, including the combination of opioid and nonopioid analgesic, regional analgesia, and nonpharmacological treatments for pain management, which the quality of their evidence is not available [4, 20, 21, 23–25]. Requirement for patient education, interprofessional collaboration, and higher doses of medication are other main findings.

Most included reviews were conducted within the past few years, indicating a recent increase in efforts to fill the knowledge gap in this field. Clinicians may benefit from this overview as it summarised and appraised currently used interventions’ quality of evidence. It helps them decide the optimal analgesia strategies based on the patient’s conditions and type of surgery. Also, this overview revealed the gaps in knowledge in the field and the necessity of designing and conducting high-quality studies. Despite the low quality of systematic reviews and their primary studies, perioperative continuation of MOUDs, particularly buprenorphine, remains clinically relevant. Implementation of individualised multimodal analgesia strategies, especially the administration of ketamine, is also highly recommended. The findings and expert opinions suggest prioritising opioid-sparing analgesics over postoperative opioids and, if opioids are needed, using them with minimum effective dose based on the patient and surgical considerations. It should be considered that tapering the postoperative opioids in this population is critical for enabling patients to return to their baseline preoperative opioid doses, but the way to achieve it remains to be demonstrated.

Australian and New Zealand College of Anaesthetists and Faculty of Pain Medicine, Acute Pain Management fifth edition, emphasises the continuation of buprenorphine perioperatively. It suggests that dividing the daily dose of buprenorphine into 2 or 3 doses may improve pain management. This guideline also recommends following the “universal precautions” for OUD patients, including multimodal analgesia, abuse-deterrent formulations, utilization of prescription drug monitoring programs, and risk management strategies. Additionally, it recommends ketamine to improve pain management in opioid-tolerant patients [27]. Recently published multiorganizational consensus from the US Health and Human Services Pain Management Best Practices Inter-Agency Task Force also recommended similar principles [28]. The UK Surgery and Opioids, Best Practice Guidelines 2021 suggest preoperative opioid users as complex cases requiring an individualized plan. This guideline recommends considering preoperative opioid weaning if feasible in selected cases [29]. A retrospective matched cohort study identified improved postoperative functional outcomes in opioid tolerant patients who reduced their preoperative morphine equivalent dose by at least 50% versus those who did not, after total joint arthroplasty [30]. These authors suggest early risk/benefit discussions with patients contemplating joint arthroplasty, with possible referral to pain specialists or primary care providers for interested patients. Otherwise, the opioid should be continued perioperatively. For patients on MOUD, an individualised plan is required. Although there is consensus that buprenorphine should be continued perioperatively, some institutions recommend a dose adjustment preoperatively for surgeries with moderate to high risk of postoperative pain [31].

### Limitations

The following potential limitations may impact the quality of this overview. Employing the systematic review filter in the search strategy may lead to losing some of the reviews which are not defined as systematic reviews but are eligible based on the criteria. Because PICO (Population, Intervention, Comparison, and Outcomes) is not clearly identified in scoping reviews, the AMSTAR2 tool may not be the ideal appraisal checklist for them. Furthermore, this overview has relied on the included reviews’ quality assessment, results, and data interpretation, which mostly have critically low methodological conduction. If complementary data were available, the results of AMSTAR2 would be more reliable. Additionally, using one reviewer instead of two independent reviewers for study selection and data extraction steps, no assessment for overlapped primary studies, and no re-assessment of quality evidence for all primary studies may limit the results of this overview.

### Future research

One of the noticeable gaps in this field is the lack of high-quality studies, in particular randomised controlled trials. Trials should consider patient-important outcomes such as quality of life and patient satisfaction, the risk of relapse of OUD, and opioid-related side effects, including depression, sedation, and respiratory suppression (Table 5). Studies with longer follow-ups to assess long-term outcomes, including morbidity and mortality, are also required. Future studies should compare various interventions and doses in the population and present data with more details to suggest the optimal doses of the MOUDs and postoperative analgesics. Furthermore, since most of the included reviews had critically low quality, there is a gap for up-to-date systematic reviews focusing on the specific interventions to capture recent studies that might be missed by the included reviews and conducted with high methodological quality. Moreover, the efficacy of nonpharmacological strategies, multimodal analgesia, and perioperative management of methadone and naltrexone should be considered.

## Conclusion

This overview showed that perioperative continuation of buprenorphine and implementation of multimodal analgesia, particularly administration of ketamine, is recommended to improve pain management and reduce opioid-related adverse effects and OUD relapse. However, most of the available systematic reviews about perioperative pain management interventions in chronic opioid users have critically low methodological quality. In the future, high-quality primary studies, especially randomised clinical trials, are required. These studies should focus on optimal analgesic doses, patients-important and long-term outcomes, and the best analgesia strategy.

## Data Availability

All data generated or analysed during this study are included in this article and no additional source data were required.

## Appendix 1

### Search strategy

**Table:** PubMed (NCBI): June 10, 2023

Search	Query	Records retrieved
#1	“pain management” OR “analgesia” OR “perioperative pain” OR “postoperative pain” OR “post-surgery pain” OR “preoperative pain” OR “pre-surgery pain” OR “after surgery pain” OR “after operation pain” OR “before surgery pain” OR “before operation pain” OR “during surgery pain” OR “during operation pain” OR “post-operative pain” OR “pre-operative pain”	500,307
#2	“opioid user*” OR “opiate user*” OR “drug user*” OR “drug abuser” OR “OUD” OR " opioid use disorder” OR “LTOT” OR “long term opioid therapy” OR “opioid tolera*” OR “chronic opioid use” OR “preoperative opioid intake” OR “opioid related disorder” OR “morphine depend*” OR “morphine abuse*” OR “heroin depend*” OR “heroin abuse*” OR “opioid depend*” OR “opiate depend*” OR “opium depend*” OR “opioid misuse*” OR “narcotic use” OR “methadone use” OR “buprenorphine use” OR “opioid addic*”	53,308
#3	#1 AND #2	4,484
	Limited to systematic review, meta-analysis, humans, English [lang]; no date limits	120

**Table:** Cochrane Database of Systematic Reviews (Ovid): June 10, 2023

Search	Query	Records retrieved
#1	“pain management” OR “analgesia” OR “perioperative pain” OR “postoperative pain” OR “post-surgery pain” OR “preoperative pain” OR “pre-surgery pain” OR “after surgery pain” OR “after operation pain” OR “before surgery pain” OR “before operation pain” OR “during surgery pain” OR “during operation pain” OR “post-operative pain” OR “pre-operative pain”	1,219
#2	“opioid user*” OR “opiate user*” OR “drug user*” OR “drug abuser” OR “OUD” OR " opioid use disorder” OR “LTOT” OR “long term opioid therapy” OR “opioid tolera*” OR “chronic opioid use” OR “preoperative opioid intake” OR “opioid related disorder” OR “morphine depend*” OR “morphine abuse*” OR “heroin depend*” OR “heroin abuse*” OR “opioid depend*” OR “opiate depend*” OR “opium depend*” OR “opioid misuse*” OR “narcotic use” OR “methadone use” OR “buprenorphine use” OR “opioid addic*”	193
#3	#1 AND #2	49
	no date limits	49

**Table:** Embase (Ovid): June 10, 2023

Search	Query	Records retrieved
#1	“pain management” OR “analgesia” OR “perioperative pain” OR “postoperative pain” OR “post-surgery pain” OR “preoperative pain” OR “pre-surgery pain” OR “after surgery pain” OR “after operation pain” OR “before surgery pain” OR “before operation pain” OR “during surgery pain” OR “during operation pain” OR “post-operative pain” OR “pre-operative pain”	308,119
#2	“opioid user*” OR “opiate user*” OR “drug user*” OR “drug abuser” OR “OUD” OR " opioid use disorder” OR “LTOT” OR “long term opioid therapy” OR “opioid tolera*” OR “chronic opioid use” OR “preoperative opioid intake” OR “opioid related disorder” OR “morphine depend*” OR “morphine abuse*” OR “heroin depend*” OR “heroin abuse*” OR “opioid depend*” OR “opiate depend*” OR “opium depend*” OR “opioid misuse*” OR “narcotic use” OR “methadone use” OR “buprenorphine use” OR “opioid addic*”	68,785
#3	#1 AND #2	6,231
	Limited to systematic review, review [publication type], humans, English [lang]; no date limits	110

**Table:** APA PsycINFO (Ovid): June 10, 2023

Search	Query	Records retrieved
#1	“pain management” OR “analgesia” OR “perioperative pain” OR “postoperative pain” OR “post-surgery pain” OR “preoperative pain” OR “pre-surgery pain” OR “after surgery pain” OR “after operation pain” OR “before surgery pain” OR “before operation pain” OR “during surgery pain” OR “during operation pain” OR “post-operative pain” OR “pre-operative pain”	24,639
#2	“opioid user*” OR “opiate user*” OR “drug user*” OR “drug abuser” OR “OUD” OR " opioid use disorder” OR “LTOT” OR “long term opioid therapy” OR “opioid tolera*” OR “chronic opioid use” OR “preoperative opioid intake” OR “opioid related disorder” OR “morphine depend*” OR “morphine abuse*” OR “heroin depend*” OR “heroin abuse*” OR “opioid depend*” OR “opiate depend*” OR “opium depend*” OR “opioid misuse*” OR “narcotic use” OR “methadone use” OR “buprenorphine use” OR “opioid addic*”	24,698
#3	#1 AND #2	1,009
	Limited to systematic review, humans, English [lang]; no date limits	0

**Table:** CINAHL (EBSCO): June 9, 2023

Search	Query	Records retrieved
#1	“pain management” OR “analgesia” OR “perioperative pain” OR “postoperative pain” OR “post-surgery pain” OR “preoperative pain” OR “pre-surgery pain” OR “after surgery pain” OR “after operation pain” OR “before surgery pain” OR “before operation pain” OR “during surgery pain” OR “during operation pain” OR “post-operative pain” OR “pre-operative pain”	69,030
#2	“opioid user*” OR “opiate user*” OR “drug user*” OR “drug abuser” OR “OUD” OR " opioid use disorder” OR “LTOT” OR “long term opioid therapy” OR “opioid tolera*” OR “chronic opioid use” OR “preoperative opioid intake” OR “opioid related disorder” OR “morphine depend*” OR “morphine abuse*” OR “heroin depend*” OR “heroin abuse*” OR “opioid depend*” OR “opiate depend*” OR “opium depend*” OR “opioid misuse*” OR “narcotic use” OR “methadone use” OR “buprenorphine use” OR “opioid addic*”	
#3	#1 AND #2	1,761
	Limited to systematic review, humans, English [lang]; no date limits	61

**Table:** Allied and Complementary Medicine (Ovid): June 10, 2023

Search	Query	Records retrieved
#1	“pain management” OR “analgesia” OR “perioperative pain” OR “postoperative pain” OR “post-surgery pain” OR “preoperative pain” OR “pre-surgery pain” OR “after surgery pain” OR “after operation pain” OR “before surgery pain” OR “before operation pain” OR “during surgery pain” OR “during operation pain” OR “post-operative pain” OR “pre-operative pain”	3,648
#2	“opioid user*” OR “opiate user*” OR “drug user*” OR “drug abuser” OR “OUD” OR " opioid use disorder” OR “LTOT” OR “long term opioid therapy” OR “opioid tolera*” OR “chronic opioid use” OR “preoperative opioid intake” OR “opioid related disorder” OR “morphine depend*” OR “morphine abuse*” OR “heroin depend*” OR “heroin abuse*” OR “opioid depend*” OR “opiate depend*” OR “opium depend*” OR “opioid misuse*” OR “narcotic use” OR “methadone use” OR “buprenorphine use” OR “opioid addic*”	489
#3	#1 AND #2	127
	Limited to review, English [lang]; no date limits	7

**Table:** Scopus (Elsevier): June 10, 2023

Search	Query	Records retrieved
#1	TITLE-ABS-KEY (“pain management” OR “analgesia” OR “perioperative pain” OR “postoperative pain” OR “post-surgery pain” OR “preoperative pain” OR “pre-surgery pain” OR “after surgery pain” OR “after operation pain” OR “before surgery pain” OR “before operation pain” OR “during surgery pain” OR “during operation pain” OR “post-operative pain” OR “pre-operative pain”)	277,024
#2	TITLE-ABS-KEY ( “opioid user*" OR “opiate user*" OR “drug user*" OR “drug abuser" OR “OUD" OR " opioid use disorder" OR “LTOT" OR “long term opioid therapy" OR “opioid tolera*" OR “chronic opioid use" OR “preoperative opioid intake" OR “opioid related disorder" OR “morphine depend*" OR “morphine abuse*" OR “heroin depend*" OR “heroin abuse*" OR “opioid depend*" OR “opiate depend*" OR “opium depend*" OR “opioid misuse*" OR “narcotic use" OR “methadone use" OR “buprenorphine use" OR “opioid addic*” )	79,586
#3	#1 AND #2	6,656
#4	(TITLE-ABS-KEY (“systematic review”))	476,352
#5	#3 AND #4	272
	Limited to systematic review, humans, English [lang]; no date limits	249

**Table:** PROSPERO/International prospective register of systematic reviews (NIHR): June 10, 2023

Search	Query	Records retrieved
#1	“pain management” OR “analgesia” OR “perioperative pain” OR “postoperative pain” OR “post-surgery pain” OR “preoperative pain” OR “pre-surgery pain” OR “after surgery pain” OR “after operation pain” OR “before surgery pain” OR “before operation pain” OR “during surgery pain” OR “during operation pain” OR “post-operative pain” OR “pre-operative pain”	4,873
#2	“opioid user*” OR “opiate user*” OR “drug user*” OR “drug abuser” OR “OUD” OR " opioid use disorder” OR “LTOT” OR “long term opioid therapy” OR “opioid tolera*” OR “chronic opioid use” OR “preoperative opioid intake” OR “opioid related disorder” OR “morphine depend*” OR “morphine abuse*” OR “heroin depend*” OR “heroin abuse*” OR “opioid depend*” OR “opiate depend*” OR “opium depend*” OR “opioid misuse*” OR “narcotic use” OR “methadone use” OR “buprenorphine use” OR “opioid addic*”	751
#3	#1 AND #2	126
	Limited to completed [status]	4

**Table:** ProQuest Dissertations & Theses A&I (ProQuest): June 10, 2023

Search	Query	Records retrieved
#1	abstract(“pain management” OR “analgesia” OR “perioperative pain” OR “postoperative pain” OR “post-surgery pain” OR “preoperative pain” OR “pre-surgery pain” OR “after surgery pain” OR “after operation pain” OR “before surgery pain” OR “before operation pain” OR “during surgery pain” OR “during operation pain” OR “post-operative pain” OR “pre-operative pain” )	4,038
#2	abstract(“opioid user*” OR “opiate user*” OR “drug user*” OR “drug abuser” OR “OUD” OR " opioid use disorder” OR “LTOT” OR “long term opioid therapy” OR “opioid tolera*” OR “chronic opioid use” OR “preoperative opioid intake” OR “opioid related disorder” OR “morphine depend*” OR “morphine abuse*” OR “heroin depend*” OR “heroin abuse*” OR “opioid depend*” OR “opiate depend*” OR “opium depend*” OR “opioid misuse*” OR “narcotic use” OR “methadone use” OR “buprenorphine use” OR “opioid addic*” )	2,739
#3	#1 AND #2	143
#4	abstract(“systematic reviews”)	1,309
#5	#3 AND #4	0
	Limited to English [lang]; no date limits	0

**Table:** Epistemonikos (https://www.epistemonikos.org): June 10, 2023

Search	Query	Records retrieved
#1	(title: (“pain management” OR “analgesia” OR “perioperative pain” OR “postoperative pain” OR “post-surgery pain” OR “preoperative pain” OR “pre-surgery pain” OR “after surgery pain” OR “after operation pain” OR “before surgery pain” OR “before operation pain” OR “during surgery pain” OR “during operation pain” OR “post-operative pain” OR “pre-operative pain”) OR abstract: (“pain management” OR “analgesia” OR “perioperative pain” OR “postoperative pain” OR “post-surgery pain” OR “preoperative pain” OR “pre-surgery pain” OR “after surgery pain” OR “after operation pain” OR “before surgery pain” OR “before operation pain” OR “during surgery pain” OR “during operation pain” OR “post-operative pain” OR “pre-operative pain”))	32,785
#2	(title: (“opioid user*” OR “opiate user*” OR “drug user*” OR “drug abuser” OR “OUD” OR " opioid use disorder” OR “LTOT” OR “long term opioid therapy” OR “opioid tolera*” OR “chronic opioid use” OR “preoperative opioid intake” OR “opioid related disorder” OR “morphine depend*” OR “morphine abuse*” OR “heroin depend*” OR “heroin abuse*” OR “opioid depend*” OR “opiate depend*” OR “opium depend*” OR “opioid misuse*” OR “narcotic use” OR “methadone use” OR “buprenorphine use” OR “opioid addic*”) OR abstract: (“opioid user*” OR “opiate user*” OR “drug user*” OR “drug abuser” OR “OUD” OR " opioid use disorder” OR “LTOT” OR “long term opioid therapy” OR “opioid tolera*” OR “chronic opioid use” OR “preoperative opioid intake” OR “opioid related disorder” OR “morphine depend*” OR “morphine abuse*” OR “heroin depend*” OR “heroin abuse*” OR “opioid depend*” OR “opiate depend*” OR “opium depend*” OR “opioid misuse*” OR “narcotic use” OR “methadone use” OR “buprenorphine use” OR “opioid addic*”))	476
#3	#1 AND #2	332
	Limited to systematic review; no date limits	84

## Appendix 2

### List of AMSTAR2 questions

Q1. Did the research questions and inclusion criteria for the review include the components of PICO?

Q2. Did the report of the review contain an explicit statement that the review methods were established prior to the conduct of the review and did the report justify any significant deviations from the protocol?

Q3. Did the review authors explain their selection of the study designs for inclusion in the review?

Q4. Did the review authors use a comprehensive literature search strategy?

Q5. Did the review authors perform study selection in duplicate?

Q6. Did the review authors perform data extraction in duplicate?

Q7. Did the review authors provide a list of excluded studies and justify the exclusions?

Q8. Did the review authors describe the included studies in adequate detail?

Q9. Did the review authors use a satisfactory technique for assessing the risk of bias (RoB) in individual studies that were included in the review?

Q10. Did the review authors report on the sources of funding for the studies included in the review?

Q11. If meta-analysis was performed did the review authors use appropriate methods for statistical combination of results?

Q12. If meta-analysis was performed, did the review authors assess the potential impact of RoB in individual studies on the results of the meta-analysis or other evidence synthesis?

Q13. Did the review authors account for RoB in individual studies when interpreting/ discussing the results of the review?

Q14. Did the review authors provide a satisfactory explanation for, and discussion of, any heterogeneity observed in the results of the review?

Q15. If they performed quantitative synthesis did the review authors carry out an adequate investigation of publication bias (small study bias) and discuss its likely impact on the results of the review?

Q16. Did the review authors report any potential sources of conflict of interest, including any funding they received for conducting the review?

## Appendix 3

**Table:** List of excluded reports in the full-text screening

Reason	Citation
Wrong study design: narrative review	Ward EN, Quaye AN, Wilens TE. Opioid Use Disorders: Perioperative Management of a Special Population. Anesth Analg. 2018 Aug;127(2):539–547. doi: 10.1213/ANE.0000000000003477. PMID: 29,847,389; PMCID: PMC6523021.
	Brooks MR, Golianu B. Perioperative management in children with chronic pain. Paediatr Anaesth. 2016 Aug;26(8):794–806. doi: 10.1111/pan.12948. PMID: 27,370,517.
	Hadi I, Morley-Forster PK, Dain S, Horrill K, Moulin DE. Brief review: perioperative management of the patient with chronic non-cancer pain. Can J Anaesth. 2006 Dec;53(12):1190-9. doi: 10.1007/BF03021580. PMID: 17,142,653.
	Prabhu M, Bortoletto P, Bateman BT. Perioperative pain management strategies among women having reproductive surgeries. Fertil Steril. 2017 Aug;108(2):200–206. doi: 10.1016/j.fertnstert.2017.06.010. Epub 2017 Jul 8. PMID: 28,697,915; PMCID: PMC5545053.
	Coluzzi F, Bifulco F, Cuomo A, Dauri M, Leonardi C, Melotti RM, Natoli S, Romualdi P, Savoia G, Corcione A. The challenge of perioperative pain management in opioid-tolerant patients. Ther Clin Risk Manag. 2017 Sep 5;13:1163–1173. doi: 10.2147/TCRM.S141332. PMID: 28,919,771; PMCID: PMC5592950.
	Lembke A, Ottestad E, Schmiesing C. Patients Maintained on Buprenorphine for Opioid Use Disorder Should Continue Buprenorphine Through the Perioperative Period. Pain Med. 2019 Mar 1;20(3):425–428. doi: 10.1093/pm/pny019. PMID: 29,452,378; PMCID: PMC6387981.
	Safley RR, Swietlikowski J. Pain Management in the Opioid-Dependent Pregnant Woman. J Perinat Neonatal Nurs. 2017 Apr/Jun;31(2):118–125. doi: 10.1097/JPN.0000000000000244. PMID: 28,437,302.
No perioperative pain management	De Aquino JP, Parida S, Avila-Quintero VJ, Flores J, Compton P, Hickey T, Gómez O, Sofuoglu M. Opioid-induced analgesia among persons with opioid use disorder receiving methadone or buprenorphine: A systematic review of experimental pain studies. Drug Alcohol Depend. 2021 Nov 1;228:109097. doi: 10.1016/j.drugalcdep.2021.109097. Epub 2021 Sep 22. PMID: 34,601,272; PMCID: PMC8595687.
	De Aquino, JP, Flores, JM, Avila-Quintero, VJ, Compton, P, Sofuoglu, M. Pharmacological treatment of pain among persons with opioid addiction: A systematic review and meta-analysis with implications for drug development. Addiction Biology. 2021; 26:e12964. 10.1111/adb.12964
	Taveros MC, Chuang EJ. Pain management strategies for patients on methadone maintenance therapy: a systematic review of the literature. BMJ Support Palliat Care. 2017 Dec;7(4):383–389. doi: 10.1136/bmjspcare-2016-001126. Epub 2016 Aug 26. PMID: 27,566,722.
Wrong population	Gallucci A, Lucena PH, Martens G, Thibaut A, Fregni F. Transcranial direct current stimulation to prevent and treat surgery-induced opioid dependence: a systematic review. Pain Manag. 2019 Jan 1;9(1):93–106. doi: 10.2217/pmt-2018-0053. Epub 2018 Dec 5. PMID: 30,516,441.
